# Dyslipidemia as a risk factor for liver fibrosis progression in a multicentric population with non-alcoholic steatohepatitis

**DOI:** 10.12688/f1000research.21918.1

**Published:** 2020-01-28

**Authors:** Nahum Méndez-Sánchez, Eira Cerda-Reyes, Fátima Higuera-de-la-Tijera, Ana K. Salas-García, Samantha Cabrera-Palma, Guillermo Cabrera-Álvarez, Carlos Cortez-Hernández, Luis A Pérez-Arredondo, Emma Purón-González, Edgar Coronado-Alejandro, Arturo Panduro, Heriberto Rodríguez-Hernández, Vania C. Cruz-Ramón, Alejandro Valencia-Rodríguez, Xingshun Qi, Nashla Hamdan-Pérez, Nancy E. Aguilar-Olivos, Beatriz Barranco-Fragoso, Oscar Ramírez-Pérez, Alfonso Vera-Barajas

**Affiliations:** 1 Liver Research Unit, Medica Sur Clinic & Foundation, Mexico City, Mexico, 14050, Mexico; 2 Faculty of Medicine, National Autonomous University of Mexico, Mexico City, Mexico; 3 Department of Gastroenterology, Central Military Hospital, Mexico City, Mexico; 4 Department of Gastroenterology, General Hospital of Mexico "Dr. Eduardo Liceaga", Mexico City, Mexico, Mexico; 5 Liver Clinic, General Regional Hospital IMSS 1, Cuernavaca, Morelos, Mexico; 6 Department of Gastroenterology, University Hospital “Jose Eleuterio González”, Monterrey, Nuevo Leon, Mexico; 7 Department of Internal Medicine, Christus Muguerza “Super Specialty Hospital”, Monterrey, Nuevo Leon, Mexico; 8 Department of Molecular Biology in Medicine, Civil Hospital of Guadalajara "Fray Antonio Alcalde”, Guadalajara, Jalisco, Mexico; 9 Faculty of Medicine and Nutrition, Juarez University of the State of Durango, Durango, Mexico; 10 Department of Gastroenterology, General Hospital of Northern Theater Command, Shenyang, Liaoning, 10016, China; 11 Department of Gastroenterology, National Medical Center "20 Noviembre", Mexico City, Mexico, 03229, Mexico

**Keywords:** non-alcoholic fatty liver disease, cirrhosis, dyslipidemia, type 2 diabetes mellitus, metabolic syndrome.

## Abstract

**Background:** Nonalcoholic fatty liver disease (NAFLD) is a serious worldwide health problem, with an estimated global prevalence of 24%; it has a notable relationship with other metabolic disorders, like obesity and type 2 diabetes mellitus (T2DM). Nonalcoholic steatohepatitis (NASH) is one of the most important clinical entities of NAFLD, which is associated with an increased risk of progression to liver cirrhosis and hepatocellular carcinoma (HCC). Mexico is one of the countries with the highest prevalence of metabolic diseases; therefore, we sought to investigate the impact that these clinical entities have in the progression to advanced fibrosis in Mexican patients with NASH.

**Methods:** We performed a multicenter retrospective cross-sectional study, from January 2012 to December 2017. A total of 215 patients with biopsy-proven NASH and fibrosis were enrolled. NASH was diagnosed according NAS score and liver fibrosis was staged by the Kleiner scoring system. For comparing the risk of liver fibrosis progression, we divided our sample into two groups. Those patients with stage F0-F2 liver fibrosis were included in the group with non-significant liver fibrosis (n=178) and those individuals with F3-F4 fibrosis were included in the significant fibrosis group (n=37). We carried out a multivariate analysis to find risk factors associated with liver fibrosis progression.

**Results:** From the 215 patients included, 37 had significant liver fibrosis (F3-4). After logistic regression analysis T2DM (p=0.044), systemic arterial hypertension (p=0.014), cholesterol (p=0.041) and triglycerides (p=0.015) were the main predictor of advanced liver fibrosis.

**Conclusions:** In a Mexican population, dyslipidemia was the most important risk factor associated with advanced liver fibrosis and cirrhosis.

## Introduction

Nonalcoholic fatty liver disease (NAFLD) has a broad clinical spectrum, ranging from simple steatosis to cirrhosis, even developing in some cases with hepatocellular carcinoma (HCC) ^1^. Nonalcoholic steatohepatitis (NASH) is one of the most important clinical entities of NAFLD, characterized by the histologic presence of liver steatosis, ballooning degeneration, and lobular inflammation, with or without fibrosis ^2^. Once NASH is established, there is a significant increased risk of developing liver cirrhosis and HCC ^3^.

Currently, NAFLD is the most common chronic liver disease in the world, with an important relationship with other metabolic disorders like obesity, type 2 diabetes mellitus (T2DM) and metabolic syndrome (MetS) ^4– 6^, being the second leading indication for liver transplantation in the United States ^7^. A global prevalence of 24% is estimated, with the highest rates in South America and the Middle East, while the lowest prevalence has been reported in Africa ^8^.

It is estimated that 30–40% of NAFLD patients will develop NASH ^9, 10^. Some studies have demonstrated that the risk of progression to liver cirrhosis in NAFLD patients is between 0–4%, while approximately 10–25% of NASH patients will develop cirrhosis ^11– 16^. This also depends on the ethnic origin of the patients, since Hispanic-Americans have been found to have a wide susceptibility to NAFLD and NASH development mainly from Mexican origin (33%) ^8^.

Multiple risk factors for NASH progression have been identified, such as diet, MetS, T2DM, obesity, Hispanic ethnicity and polymorphisms in the patatin-like phospholipase domain-containing 3 ( *PNPLA3*) gene ^17– 19^. However, the pathological mechanisms, by which some NAFLD patients progress to NASH are still not well understood ^20^.

Mexico is one of the countries with the highest prevalence of metabolic diseases; 75.2% of the Mexican population are obese or overweight, 10.3% have T2DM and 19.5% have dyslipidemia ^21^. We therefore sought to investigate the main metabolic factors involved in the progression to advanced fibrosis in Mexican patients with NASH.

## Methods

### Study design

We conducted a multicenter retrospective cross-sectional study from January 2012 to December 2017 in 7 tertiary referral centers from different parts of Mexico: Medica Sur Clinic and Foundation (Mexico City), General Hospital of Mexico “Dr. Eduardo Liceaga” (Mexico City), Civil Hospital of Guadalajara “Fray Antonio Alcalde” (Jalisco), Christus Muguerza “Super Speciality” Hospital (Nuevo Leon), Central Military Hospital (Mexico City), General Hospital of the Mexican Social Security Institute (Durango), and the General Regional Hospital, IMSS 1 (Morelos).

This study was reviewed and approved by the Ethics Committee of the Medica Sur Clinic and Foundation. Patients were not required to give informed consent to the study because the analysis used anonymous clinical data.

### Patients and data collection

We included patients older than 20 years, of both genders, who had the diagnosis of biopsy-proven NASH. NASH was diagnosed according to NAS score ^5, 6^, and liver fibrosis was staged by the Kleinier scoring system ^15^. We reviewed the medical records to obtain the baseline characteristics, biochemical parameters and comorbidities excluding all patients with other causes of liver disease, such as alcoholic liver disease, hepatitis B virus, hepatitis C virus, autoimmune liver disease, iron overload, drug-induced liver disease, and hereditary liver disease.

Several variables that have been commonly associated as risk factors for NASH progression were studied and included in the statistical analyses, such as MetS diagnosed with three or more of the following criteria established by the National Cholesterol Education Program (NCEP) Adult Treatment Panel III (ATP III) ^22^: waist circumference over 102 cm (men) or 88 cm (women), blood pressure over 130/85 mmHg, fasting triglyceride (TG) level over 150 mg/dl, fasting high-density lipoprotein (HDL) cholesterol level less than 40 mg/dl (men) or 50 mg/dl (women) and fasting blood glucose over 100 mg/dl. To define overweight or obesity we use the body mass index (BMI) defined as weight (in kg) / height ^2^ (in m ^2^). Where overweight is defined above 25 kg / m ^2^, and obesity as > 30 kg / m ^2^. T2DM was diagnosed according to the history of diseases, fasting glucose (>126 mg/dL), and/or glucose tolerance test (>200 mg/dL two hours after the glucose load). Systemic arterial hypertension was diagnosed according to the history of diseases and/or intake of antihypertensive drugs. Cardiovascular disease (CVD) was defined as the presence of history such as systemic arterial hypertension, coronary heart disease, previous heart attack or stroke. Laboratory evaluation in all patients included serum liver tests (aspartate transaminase, alanine transaminase, total bilirubin, alkaline phosphatase, and albumin), hemoglobin, platelet count, cholesterol, low-density lipoprotein, HDL, TG, and blood glucose. The diagnosis of HCC was based on histopathological findings by liver biopsy.

For comparing the risk of liver fibrosis progression, we divided our sample into two groups. Those patients with stage F0-F2 liver fibrosis were included in the group with non-significant liver fibrosis (n=178) and the individuals with stage F3-F4 fibrosis were included in the significant fibrosis group (n=37).

### Statistical analyses

We produced descriptive statistics for baseline patient characteristics. Univariate analysis of quantitative variables was conducted using Student’s t-test, while for qualitative variables we used the Chi square test. Multivariate regression analyses were used to assess the independent association between significant fibrosis and possible risk factors. A p-value <0.05 was determined to indicate significance. All analyses were performed using STATA (v14.1, Stata Corp, College Station, TX, USA).

## Results

We included 215 patients ( Table 1). Non-significant fibrosis (F0-F2) was found in 83% of our sample; 7% had fibrosis at stage F-3, and 10% had fibrosis at stage F-4. Five patients were diagnosed with HCC ( Table 2). De-identified individual-level results are available as *Underlying data* ^23^.

**Table 1.** Patient characteristics.

Variable	Result
Age (years), mean (SD)	51.9 (13.4)
Female, n (%)	140 (65)
Hemoglobin (g/dL), mean (SD)	13.8 (2.1)
Platelet count (per μL), mean (SD)	215.6 (101.2)
Cholesterol (mg/dL), mean (SD)	185 (51.3)
Low density lipoprotein (mg/dL), mean (SD)	111.2 (4.9)
High density lipoprotein (mg/dL), mean (SD)	42.8 (11.0)
Triglycerides (mg/dL), mean (SD)	162.5 (61.2)
Blood glucose (mg/dL), mean (SD)	116.4 (47.6)
Albumin (g/dL), mean (SD)	3.7 (0.6)
Total bilirubin (mg/dL), mean (SD)	1.6 (2.5)
Alanine transaminase (U/L), mean (SD)	71.8 (116.9)
Aspartate transaminase (U/L), mean (SD)	83.5 (144.8)
Alkaline phosphatase (U/L), mean (SD)	169.2 (160.7)
Body mass index (kg/m ^2^), mean (SD)	29.1 (4.9)
Type 2 diabetes mellitus, n (%)	76 (35)
Systemic arterial hypertension, n (%)	68 (32)
Metabolic syndrome, n (%)	65 (30)
Cardiovascular disease, n (%)	18 (8)
Hepatocellular carcinoma, n (%)	4 (2)

**Table 2.** Histopathological characteristics.

NAS Score items	Result
Steatosis score, n (%)	
0	1 (0.5)
1	67 (31)
2	117 (53)
3	30 (14)
Ballooning score, n (%)	
0	0 (0)
1	71 (33)
2	143 (66)
3	1 (0.5)
Inflammation score, n (%)	
0	1 (0.5)
1	75 (35)
2	117 (55)
3	22 (10)
Fibrosis stage, n (%)	
F0	133 (62)
F1	32 (15)
F2	13 (6)
F3	16 (7)
F4	21 (10)

### Prediction of significant fibrosis stage and liver cirrhosis

Compared to the group with non-significant liver fibrosis (stages F0, F1 and F2), those with significant fibrosis (stages F3 or F4) were more likely to be older and have lower levels of platelet count and albumin ( Table 3 and Table 4). Moreover, those with significant fibrosis showed higher levels of blood glucose and proportions of dyslipidemia, T2DM, MetS, systemic arterial hypertension, and CVD.

**Table 3.** Difference between fibrosis stages 0-2 versus stages 3-4.

Variables	F0-2 (n= 178)	F3-4 (n= 37)	P-value
Age (years)	50.8 ±13.9	56.7 ±9.6	0.011
Female n (%)	115 (63.5%)	30 (73.27%)	0.242
Hemoglobin (g/dL) mean ± SD	13.7 ±2.2	14.3 ±1.7	0.091
Platelet count (per μL) mean ± SD	232.7 ±98.9	142.6 ±76.5	<0.001
Cholesterol (mg/dL) mean ± SD	167 ±47.9	189 ±51.3	0.013
Low density lipoprotein (mg/dL) mean ± SD	105.9 ±28.3	112.5 ±23.9	0.126
High density lipoprotein (mg/dL) mean ± SD	43.6 ±10.6	38.2 ±12	0.004
Triglycerides (mg/dL) mean ± SD	137.7 ±66.7	168.1 ±58.6	0.004
Blood glucose (mg/dL) mean ± SD	114.6 ±45.7	136.8 ±52.3	0.007
Albumin (g/dL) mean ± SD	3.7 ±0.5	3.4 ±0.8	0.004
Total bilirubin (mg/dL) mean ± SD	1.4 ±1.7	1.3 ±0.8	0.698
Alanine transaminase (U/L) mean ± SD	67.7 ±68.3	51.1 ±36.1	0.132
Aspartate transaminase (U/L) mean ± SD	71.1 ±79	66.3 ±48	0.709
Body mass index mean ± SD	28.9 ±4.8	30.2 ±4.9	0.121
Type 2 diabetes mellitus n (%)	52 (28.7%)	24 (58.5%)	<0.001
Metabolic syndrome n (%)	49 (27.1%)	21 (51.2%)	0.003
Systemic arterial hypertension n (%)	47 (26.0%)	23 (56.1%)	<0.001
Cardiovascular disease n (%)	12 (7%)	6 (16%)	0.094

**Table 4.** Difference between fibrosis stage 0-3 versus 4.

Variables	F0-3 (n= 194)	F4 (n= 21)	P-value
Age (years)	51.1 ±13.5	58.0 ±10	0.017
Female n (%)	129 (65.2%)	16 (66.7%)	0.883
Hemoglobin (g/dL) mean ± SD	13.7 ±2.1	14.5 ±1.6	0.092
Platelet count (per μL) mean ± SD	228 ±99	115 ± 56	0.000
Cholesterol (mg/dL) mean ± SD	163 ±47	188 ±51	0.026
Low density lipoprotein (mg/dL) mean ± SD	99.7 ±31	113 ±24	0.015
High density lipoprotein (mg/dL) mean ± SD	43.3 ±10.7	37.5 ±12.3	0.015
Triglycerides (mg/dL) mean ± SD	122.1 ±63.9	167.4 ±59.2	0.001
Blood glucose (mg/dL) mean ± SD	115.3 ±45.5	146.2 ±56.2	0.003
Albumin (g/dL) mean ± SD	3.7 ±0.5	3.2 ±0.8	0.000
Total bilirubin (mg/dL) mean ± SD	1.3 ±1.7	1.5 ±0.9	0.543
Alanine transaminase (U/L) mean ± SD	66.1 ±66.4	52.7 ±35.3	0.334
Aspartate transaminase (U/L) mean ± SD	69.5 ±76.4	76.3 ±52.8	0.670
Body mass index mean ± SD	29 ±4.8	30.9 ±5.4	0.071
Metabolic syndrome n (%)	57 (28.8%)	13 (54.2%)	0.012
Type 2 diabetes mellitus n (%)	60 (30.3%)	16 (66.7%)	<0.001
Systemic arterial hypertension n (%)	60 (30.3%)	10 (41.7%)	0.258
Cardiovascular disease n (%)	13 (6.7%)	5 (24%)	0.020

Logistic multivariate regression analysis demonstrated that T2DM, systemic arterial hypertension, high cholesterol and hypertriglyceridemia significantly increased the risk for advanced liver fibrosis ( Table 5), but only T2DM and hypertriglyceridemia significantly raised the risk of developing cirrhosis. ( Table 6).

**Table 5.** Logistic regression analysis of predictors of advanced liver fibrosis stage 3-4 (n=37).

Variables	Crude OR			Adjusted OR		
	OR	95%CI	P value	OR	95%CI	P value
Age (years)	1.04	1.01-1.07	0.012			
Female	0.64	0.30-1.36	0.244			
Hemoglobin (g/dL)	1.161	0.98-1.38	0.093			
Platelet count (per μL)	0.99	0.98-0.99	<0.001	0.98	0.98-0.99	<0.001
Cholesterol (mg/dL)	2.27	1.13-4.56	0.009	2.56	1.04-6.31	0.041
Low density lipoprotein (mg/dL)	1.41	1.03-1.93	0.008			
High density lipoprotein (mg/dL)	0.81	0.71-1.61	0.223			
Triglycerides (mg/dL)	1.78	1.18-2.70	0.001	2.55	1.20-5.39	0.015
Blood glucose (mg/dL)	1.01	1.01-1.02	0.010	1.01	1.00-1.02	0.056
Albumin (g/dL)	0.46	0.27-0.79	0.005	0.41	0.19-0.89	0.024
Total bilirubin (mg/dL)	0.95	0.75-1.20	0.698			
Alanine transaminase (U/L)	0.99	0.98-1.00	0.142			
Aspartate transaminase (U/L)	0.99	0.99-1.00	0.708			
Body mass index	1.05	0.98-1.12	0.125			
Type 2 diabetes mellitus	4.60	1.86-11.32	<0.001	2.34	1.02-5.37	0.044
Metabolic syndrome	2.92	1.23-6.90	0.012			
Systemic arterial hypertension	1.64	0.69-3.90	0.258	2.59	1.21-5-55	0.014
Cardiovascular disease	3.74	1.20-11.64	0.016			

**Table 6.** Logistic regression analysis of predictors of cirrhosis stage 4 (n=21).

Variables	Crude OR			Adjusted OR		
	OR	95%CI	P value	OR	95%CI	P value
Age (years)	1.04	1.00-1.08	0.019			
Sex	0.93	0.38-2.29	0.883			
Hemoglobin (g/dL)	1.21	0.96-1.52	0.094			
Platelet count (per μL)	0.98	0.97-0.98	0.000	0.98	0.97-0.99	0.001
Cholesterol (mg/dL)	2.21	0.88-5.51	0.050			
Low density lipoprotein (mg/dL)	1.92	1.14-3.25	0.009	3.04	1.19-7.78	0.020
High density lipoprotein (mg/dL)	0.94	0.72-1.24	0.117			
Triglycerides (mg/dL)	3.10	1.41-6.81	0.000	4.96	1.69-14.48	0.003
Blood glucose (mg/dL)	1.01	1.00-1.01	0.005	1.01	0.99-1.02	0.059
Albumin (g/dL)	0.31	0.16-0.59	0.000	0.28	0.10-0.77	0.014
Total bilirubin (mg/dL)	1.06	0.86-1.32	0.546			
Alanine transaminase (U/L)	0.99	0.98-1.00	0.339			
Aspartate transaminase (U/L)	1.00	0.99-1.00	0.670			
Body mass index	1.07	0.99-1.15	0.075			
Metabolic syndrome	2.92	1.23-6.90	0.012			
Type 2 diabetes mellitus	4.60	1.86-11.32	0.001	4.53	1.49-13.82	0.008
Systemic arterial hypertension	1.64	0.69-3.90	0.258			
Cardiovascular disease	3.74	1.20-11.64	0.016			

## Discussion

In this study we found that T2DM, triglycerides and cholesterol were the main factors associated with advanced fibrosis stages and cirrhosis. In this line, the association between T2DM and NASH progression has been widely demonstrated in a large number of studies from different geographical points ^24– 28^. Our findings support this association in patients of Mexican origin. On the other hand, in recent years some experimental and clinical studies have suggested an important role of lipid deregulation in the progression of fibrosis. Even though hepatic accumulation of triglycerides is the main feature of NAFLD, it has only been related with the development of simple steatosis due to its low lipotoxic characteristics. Contrarily, free cholesterol (FC) is an important lipotoxic species commonly found in NASH patients that has been linked to hepatic inflammation and fibrosis ^29^.

The first cholesterol-induced NASH hypothesis was proposed more than 10 years ago by suggesting that mitochondrial FC loading changed the fluidity of the mitochondrial membranes, leading to the oxidation of mitochondrial glutathione, and sensitized hepatocytes to tumor necrosis factor and Fas-dependent death signaling via mitochondrial glutathione depletion, exacerbating NASH ^30^. Furthermore, hyperinsulinemia (another characteristic usually found in NASH) and cholesterol accumulation was seen to upregulate sterol regulatory element-binding protein 2 (SREBP-2), leading to increased cholesterol synthesis and downregulation of mitochondrial β-oxidation, with the consequent accumulation of cholesterol and free fatty acids in the liver ^31^. All these events precipitate hepatocyte injury, apoptosis, macrophage recruitment, liver fibrosis, and progression from steatosis to NASH ^32^.

More recent evidence has found that when feeding C57BL/6 mice with a high cholesterol + methionine/choline-deficient diet for 12 weeks, there was an increase in the development of fibrosis due to the intrahepatic accumulation of FC in the hepatic stellate cells (HSCs) via TLR4 activation with the consequent downregulation of the transforming growth factor-beta (TGF-β) pseudoreceptor bone morphogenetic protein and activin membrane-bound inhibitor, leading to TGF-β-induced fibrosis ^33^.

In addition, FC was found to alter the SREBP2-mediated feedback system, likely due to an increase in the expression of the SREBP cleavage-activating protein (SCAP) and the lack of expression of insulin induced gene 2 (Insig-2) in HSCs which favored the accumulation of cholesterol and the transdifferentiation of HSCs into myofibroblasts ^33^.

In humans, a retrospective study conducted in cirrhotic patients found that cholesterol was a strong mortality predictor in those patients ^34^. Also, in a more recent study, a group of NAFLD surgical specimens was studied observing an accumulation of FC and oxidized low-density lipoprotein in the portal vein and the hepatic sinusoidal parenchyma in the form of cholesterol crystals, related to the activation of NOD-like receptor family pyrin domain containing protein 3 (NLRP3) inflammasome promoting inflammation, HSCs activation and a characteristic chicken-wire fibrosis ^35^. Furthermore, basic research has demonstrated that cholesterol crystals (lipid droplets with a strong birefringence under polarized light) are only present in NASH-models (not in simple steatosis). Under these conditions, activated Kupffer cells (KCs) aggregate along cholesterol crystals forming “crown-like structures” intimately related to the development of foam cells and therefore atherosclerosis ^36^.

The latter demonstrates the importance of CVD with NASH, being the first cause of death in this group of patients. Therefore, our findings demonstrate the important relation that metabolic diseases have with advanced stages of NASH. This will lead to the necessity of better preventive programs with a greater impact and coverage for general population around the world.

Finally, we must point out the limitations of this study such as the cross-sectional design which precluded the establishment of causal and temporal relationships between NASH progression and associated variables. Also, the small number of patients with significant fibrosis was important to mention.

In conclusion, our results show that dyslipidemia is one of the main risk factors associated with the development of significant liver fibrosis stages. Hispanics/Latinos and the Mexican population have one of the highest rates of metabolic diseases around the world; for this reason, it is expected that NAFLD and NASH will be the leading cause of cirrhosis in the near future. Therefore, it could be crucial to develop better screening programs for NAFLD patients with hypercholesterolemia and/or another component of MetS in order to prevent the development of more significant clinical complications.

## Data availability

### Underlying data

Harvard Dataverse: Dyslipidemia as a risk factor for liver fibrosis progression in a multicentric population with non-alcoholic steatohepatitis. https://doi.org/10.7910/DVN/EE3DIC ^23^.

This project contains de-identified data for each patient assessed in this study.

Data are available under the terms of the Creative Commons Zero “No rights reserved” data waiver (CC0 1.0 Public domain dedication).
