# Supplementary material for: Dyslipidemia as a risk factor for liver fibrosis progression in a multicentric population with non-alcoholic steatohepatitis
Source: F1000Res. 2020 Jan 28;9:56. [Version 1] doi: 10.12688/f1000research.21918.1 (PMC7308903; doi:10.12688/f1000research.21918.1)
Supplement: Peer review history

# Peer review: Reviewer response for version 1

**DOI:** 10.5256/f1000research.24167.r59240

**Published:** 2020-06-16

**Authors:** Piero Portincasa

**Affiliations:** 1 Division of Internal Medicine, Clinica Medica A. Murri, Department of Biomedical Sciences and Human Oncology, AOU Policlinico di Bari, University of Bari Aldo Moro, Bari, Italy

This is a multicentre retrospective cross-sectional study on NAFLD (2012-17) with 215 patients undergoing liver histology (NASH-fibrosis). Group F0-F2 fibrosis and Group F3-F4 were compare by logistic regression analysis. T2DM,  systemic arterial hypertension, cholesterol and triglycerides  were the main predictor of advanced liver fibrosis

- The paper is well presented and simple in the take-home message. The authors conclude that T2DM, serum triglycerides and cholesterol were the main factors associated with advanced fibrosis stages and cirrhosis.
- A general point is to discuss the limited n. of patients with fibrosis (N=37) and how this number could affect the overall results of the study. This aspect is only briefly touched at the end of discussion.
- Also, can the authors provide any information about pre-T2DM, i.e. the presence of insulin resistance?
- Abstract: please better describe the terms “cholesterol” and “triglycerides” (serum chol, TG)
- Intro: the authors should also mention the recent consensus paper in gastro re-classifying the metabolic NAFLD Eslam M, Sanyal AJ, George J, International Consensus Panel. MAFLD: A Consensus-Driven Proposed Nomenclature for Metabolic Associated Fatty Liver Disease ^1^.
- When mentioning PNPLA3, please refer to a previous paper Krawczyk M, Portincasa P, Lammert F. PNPLA3-associated steatohepatitis: toward a gene-based classification of fatty liver disease ^2^.
- Table 1: The table should bring the total number of enrolled patients at the top. I would suggest to simplify by mentioning at the bottom: data are mean (SD)
- Discussion: The study is only observational and not longitudinal (till 2017). Can the authors provide info about the follow-up of some of these patients with advanced fibrosis?
- I also would spend few words about the role of therapeutic agents (hypolipidemic drugs) on limiting the progression of NASH

Is the work clearly and accurately presented and does it cite the current literature?

Yes

If applicable, is the statistical analysis and its interpretation appropriate?

Yes

Are all the source data underlying the results available to ensure full reproducibility?

Partly

Is the study design appropriate and is the work technically sound?

Yes

Are the conclusions drawn adequately supported by the results?

Partly

Are sufficient details of methods and analysis provided to allow replication by others?

Yes

Reviewer Expertise:

Internal medicine, metabolic disorders, lipidology, hepatology

I confirm that I have read this submission and believe that I have an appropriate level of expertise to confirm that it is of an acceptable scientific standard.

## References

[1] : MAFLD: A Consensus-Driven Proposed Nomenclature for Metabolic Associated Fatty Liver Disease. *Gastroenterology*.2020;158(7) : 10.1053/j.gastro.2019.11.312 1999-2014.e1 10.1053/j.gastro.2019.11.312 32044314

[2] : PNPLA3-associated steatohepatitis: toward a gene-based classification of fatty liver disease. *Semin Liver Dis*.2013;33(4) : 10.1055/s-0033-1358525 369-79 10.1055/s-0033-1358525 24222094

# Peer review: Reviewer response for version 1

**DOI:** 10.5256/f1000research.24167.r59237

**Published:** 2020-02-18

**Authors:** Kevork Peltekian

**Affiliations:** 1 Division of Digestive Care & Endoscopy, Atlantic Multi-Organ Transplantation Program, QEII Health Sciences Centre, Dalhousie University, Halifax, NS, Canada

The authors should be congratulated for prospectively collecting information regarding risk factors for advanced liver fibrosis in patients with non-alcoholic steatohepatitis. The medical literature lacks enough data. The main predictors obviously include thrombocytopenia and components of metabolic syndrome.

 The authors may want to discuss the metabolic syndrome is an independent factor for predicting more advanced fibrosis (if the statistics confirms it).

 The discussion can be simplified by minimizing results of animal studies and enhancing more results of human studies.

Is the work clearly and accurately presented and does it cite the current literature?

Yes

If applicable, is the statistical analysis and its interpretation appropriate?

Yes

Are all the source data underlying the results available to ensure full reproducibility?

Yes

Is the study design appropriate and is the work technically sound?

Yes

Are the conclusions drawn adequately supported by the results?

Yes

Are sufficient details of methods and analysis provided to allow replication by others?

Yes

Reviewer Expertise:

Hepatology

I confirm that I have read this submission and believe that I have an appropriate level of expertise to confirm that it is of an acceptable scientific standard.

# Response

**Published:** 2020-02-18

**Authors:** Nahum Méndez-Sánchez

**Affiliations:** Medica Sur Clinic and Foundation, Mexico

Dear Dr. Peltekian,

 We appreciate your comments and we are honored to have had the opportunity of being evaluated by a distinguished member of the hepatology field. We agree that not all animal studies are reproducible in humans, however, we believe that to support our results it is important to include them since currently there is an important lack of information in humans about dyslipidemia as a risk factor for liver fibrosis progression.
